# A Catheter-Related Bloodstream Infection by *Brevibacterium casei* in a Child with Acute Myeloid Leukemia: Case Report and Literature Review

**DOI:** 10.1155/2021/6691569

**Published:** 2021-04-09

**Authors:** Fumihiro Ochi, Hisamichi Tauchi, Kyoko Moritani, Shinobu Murakami, Hitoshi Miyamoto, Mayo Ueda, Kozo Nagai, Minenori Eguchi-Ishimae, Mariko Eguchi

**Affiliations:** ^1^Department of Pediatrics, Ehime University Graduate School of Medicine, Toon, Ehime 791-0295, Japan; ^2^Clinical Laboratory Division, Ehime University Hospital, Toon, Ehime 791-0295, Japan

## Abstract

The most common organisms isolated from pediatric catheter-related bloodstream infections (CRBSIs) are Gram-positive cocci, such as coagulase-negative staphylococci and *Staphylococcus aureus*. There are few formal reports of *Brevibacterium casei* infection and even fewer reports of CRBSI due to this Gram-positive rod. Here we report the first case of CRBSI due to *B. casei* in an 8-year-old girl with acute myeloid leukemia in Japan. The isolate exhibited decreased susceptibility to *ß*-lactam antibiotics. Antimicrobial therapy with meropenem and vancomycin, in addition to the removal of central venous catheter line, consequently led to a significant clinical improvement of the patient's symptoms. A literature review found available clinical courses in 16 cases (4 pediatric cases including our case) of *B. casei* infection. Our case and those in literature suggested that *B. casei* infection often occurs in patients with indwelling central venous catheters; the literature review further suggested that removal of central venous catheters is required in most cases. Special attention should be paid to the detection of opportunistic infections due to *Brevibacterium* spp. in immunocompromized children who are using a central venous catheter.

## 1. Introduction

There are only a few formal reports on *Brevibacterium casei* infections, especially in immunocompromized children. We report the first case of catheter-related bloodstream infection (CRBSI) due to *B. casei* in a child with acute myeloid leukemia (AML) in Japan.

Chemotherapy in the treatment of AML induces long-term neutropenia, which greatly increases the risk of infection. In addition, there is also a high risk of healthcare-associated infections, such as CRBSI, due to the need for long-term central venous catheterization.

*B. casei* is an obligately aerobic, catalase-positive, non-spore-forming, immotile, Gram-positive rod and a known human skin colonizer [1]. *B. casei* was not considered a human pathogen until the publication of a few reports of infections in immunocompromized patients. Recently, *B. casei* has emerged as an opportunistic pathogen in immunocompromized hosts and has been associated with severe infections, such as bacteremia, brain abscess, pericardial infection, peritonitis, and endophthalmitis [2–15]. Previous studies have shown blood cultures to be the most common specimens from which *B. casei* was isolated. Reports of CRBSI due to *B. casei* in immunocompromized hosts are on the rise [16].

Currently, there are no large studies investigating appropriate antibiotics or treatment duration for *B. casei* infections. We therefore conducted a literature review to find an appropriate treatment. We summarized the previously reported cases of *B. casei* infection by performing a PubMed search from January 1995 to March 2020 (Table 1). Among the 16 patients with reported *B. casei* infections, 4 were pediatric patients (<15 years old), including our patient [2–15]. This report aims to present a case report of a CRBSI caused by a *B. casei* infection, review previous *B. casei* infections, and provide a concise review of the clinical background, risk factors, and management of infections due to this organism.

## 2. Case Report

An 8-year-old girl diagnosed with AML (standard risk) was transferred to our hospital. According to the AML12 protocol of the Japanese Pediatric Leukemia/Lymphoma Study Group (JPLSG), she received combination chemotherapy (cytarabine, methotrexate, mitoxantrone, idarubicin, and etoposide) as induction therapy and achieved complete remission. Neutropenia became apparent 6 days after maintenance therapy, with neutrophil counts of <500/*μ*L, and the patient developed febrile episodes with shaking chills 9 days after maintenance therapy. She appeared toxic, and her temperature, blood pressure, pulse rate, and respiratory rate were 38.7°C, 88/48 mmHg, 108/min, and 24/min, respectively.

Laboratory examination revealed a decreased leukocyte count of <100/*μ*L and elevated C-reactive protein level of 3.58 mg/dL (reference range, <0.5 mg/dL). After two consecutive blood cultures taken from the peripheral vein and peripherally inserted central catheter (PICC) line, meropenem (40 mg/kg/dose, 3 times/day) was administered as empiric therapy. The standard blood culture exhibited only coryneform Gram-positive, club-shaped, slightly curved rods from the aerobic bottle 1 day after culture. Her temperature, blood pressure, pulse rate, and respiratory rate were 36.7°C, 76/40 mmHg, 96/min, and 18/min, respectively. We then added vancomycin (15 mg/kg/dose, 4 times/day) to the treatment protocol and removed the PICC line on the second day of the febrile episode. The duration of PICC placement was 69 days.

Using a MALDI Biotyper (Bruker Daltonik GmbH, Bremen, Germany) with laser desorption ionization time-of-flight mass spectrometry, we identified *B. casei* in the positive blood cultures containing samples taken from the peripheral vein and PICC line [17]. *B. casei* also grew in the sample collected from the PICC tip, based on which we made a diagnosis of CRBSI caused due to *B. casei*.

**Figure 1:**
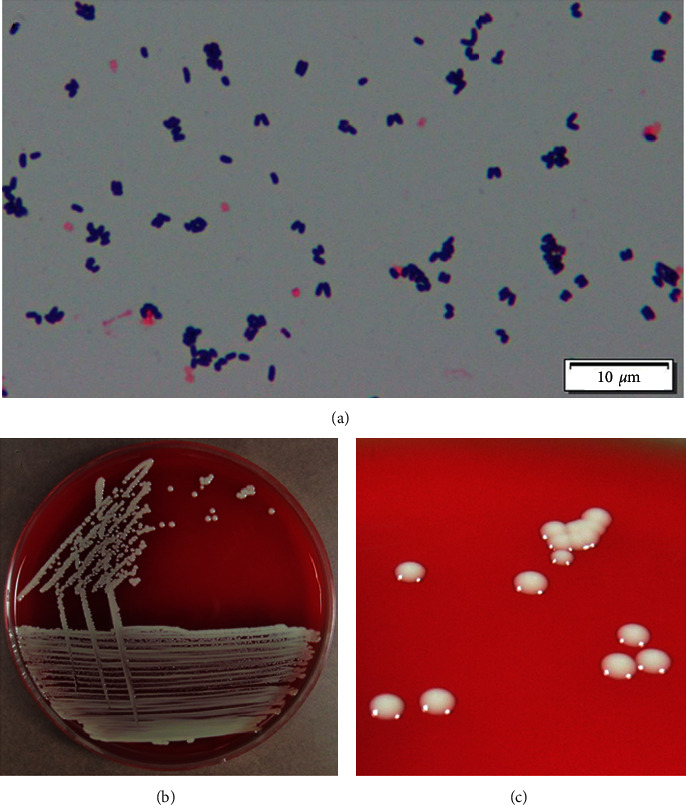
Macroscopic and microscopic appearance of *Brevibacterium casei*. (a) Gram-stained smears from blood samples cultured in bottles at 37°C for 24 h. (b) Macroscopic appearance of *B. casei* colonies after isolation from the patient and culture on a sheep blood agar plate incubated at 37°C for 24 h in a CO_2_ atmosphere. (c) The colonies appeared gray-white in color, non-hemolytic, smooth, and round and had a distinctive cheese odor.

After a 24 h incubation at 37°C in a CO_2_ atmosphere, the Gram-positive rods formed colonies on sheep blood agar that were whitish to gray-white in color, non-hemolytic, smooth, and round and had a distinctive cheese odor (Figure 1). The isolate was positive for catalase, *α*-glucosidase, and gelatin hydrolysis, while negative for urease, oxidase, and nitrate reduction. These biochemical features were typical of *B. casei*. Drug sensitivity tests were analyzed using the broth microdilution method. The minimum inhibitory concentration for the isolate was >4 *μ*g/mL for ampicillin, sulbactam/ampicillin, and tazobactam/piperacillin; >2 *μ*g/mL for cefazolin and cefmetazole; ≤2 *μ*g/mL for cefepime; 0.25 *μ*g/mL for meropenem; 2 *μ*g/mL for clarithromycin; 4 *μ*g/mL for ciprofloxacin; and 0.5 *μ*g/mL for vancomycin.

There is no standardized treatment for *B. casei* bacteremia, and the Clinical and Laboratory Standards Institute 2011 criteria for interpreting susceptibility results are based on the recommendations that apply to *Corynebacterium* spp. [18]. In our case, antimicrobial therapy with meropenem and vancomycin and PICC line removal consequently led to a significant clinical improvement of the symptoms. We performed antimicrobial treatment for 19 days until the patient's neutropenia was ameliorated, and she recovered without sequelae. After the chemotherapy as treatment of AML, the patient remained in remission. At present, she is undergoing close follow-up as an outpatient.

## 3. Discussion

The genus *Brevibacterium* consists of 45 species, of which only 10 have been isolated from clinical samples (*B. avium*, *B. casei*, *B. epidermidis*, *B. iodinum*, *B. linens*, *B. massiliense*, *B. mcbrellneri*, *B. otitidis*, *B. paucivorans*, and *B. sanguinis*). *B. casei* is the most frequently isolated *Brevibacterium* species from otherwise sterile human sites [19].

Most patients with *B. casei* infection presented with specific underlying conditions, such as malignant tumors, renal failure, or an immunocompromized status (Table 1). Our patient presented with AML. Medical catheters are often required for treatment in patients with underlying diseases such as those mentioned. Patients with indwelling central venous catheters are at high risk of acquiring CRBSIs.

The most common organisms isolated from pediatric CRBSIs are coagulase-negative staphylococci and *Staphylococcus aureus*. Although *B. casei* is an extremely rare organism isolated from patients with CRBSI, it is crucial to be aware of the possibility of *B. casei* infection in immunocompromized hosts with catheter devices because central venous catheters and peritoneal dialysis catheters were the most common causes of *B. casei* infections (Table 1).

Interestingly, the *B. casei* isolates exhibited varying degrees of susceptibility to a variety of antimicrobial agents (Table 1). Although most isolates had *β*-lactam minimum inhibitory concentrations that fell within the susceptible range, some exhibited decreased susceptibility to all of the *β*-lactam antibiotics, such as in our case. Accordingly, the inclusion of glycopeptide administration in the definitive therapy is recommended. Almost all reported *B. casei* CRBSI cases were treated with glycopeptides.

In general, CRBSI management consists of systemic antibiotic therapy and catheter removal (if feasible). Catheter removal, in addition to systemic antimicrobial therapy administration, is recommended in circumstances such as sepsis, endocarditis, metastatic infection, thrombophlebitis, persistent bacteremia, subcutaneously tunneled central venous catheter infection, or port reservoir infection due to the high likelihood of severe and/or progressive infection with antibiotic therapy alone [20]. For children with CRBSI, some pediatricians favor attempting catheter salvage, such as antibiotic lock therapy, when feasible, given the greater difficulty of vascular access among children than among adults.

Five patients with CRBSI due to *B. casei* received antibiotic therapy without catheter removal (Nos. 2, 6, 7, 14, and 15), and four out of five patients with *B. casei* infection and no catheter removal had a relapsed infection. Conversely, in patients with CRBSI due to *B. casei* in whom catheter removal was performed as empiric therapy (0/4 patients), relapsed infections did not occur (Table 1). We performed both antibiotic administration and catheter removal in our case, and our patient recovered without sequelae. Thus, the removal of infected or unnecessary catheters, if possible, is desirable.

In conclusion, special attention should be paid to opportunistic infections due to *Brevibacterium* spp. in immunocompromized children who are using a central venous catheter. It is crucial to minimize the risk of infection from contaminated sources (needleless connectors, catheter hubs, or injection ports) and to remove infected or unnecessary catheters.

## Figures and Tables

**Table 1:** Characteristics, treatment, and outcomes of the *Brevibacterium casei* infection cases identified in a PubMed search conducted in March 2020.

No	Age (y), sex	Underlying disease	Infection	Device	Empiric therapy (days^‡^)	Treatment after relapse (days^‡^)	Ref.
1	25, M	Choriocarcinoma	Sepsis	Permanent catheter	PIPC + TEIC (10)	PIPC + TOB (10)	[2]
2	46, F	NHL	CRBSI	CVC	CEX (10)	Device removal	[3]
3	N/A^∗^	Neuroblastoma	CRBSI	Broviac catheter	N/A	N/A	[4]
4	18, F	AIDS	Sepsis, CRBSI	Port-a-cath	CPFX (14) + device removal	No relapse	[5]
5	34, M	AIDS	Sepsis, CRBSI	Hickman catheter	CAZ + VCM (8) + device removal	No relapse	[6]
6	43, F	Crohn's disease	CRBSI	Port-a-cath	VCM (15)	CVA/AMPC (3), MEPM (3), VCM (3) + device removal	[7]
7	31, M	N/A, HD	CRBSI	Hickman catheter	VCM (15)	VCM + antibiotic lock (15)	[7]
8	78, M	Cancer	Pericardial infection	N/A	VCM (11) + Pericardiocentesis	No relapse	[8]
9	62, F	PH	Sepsis, CRBSI	CVC	MFLX (21) + VCM (10) + device removal	No relapse	[9]
10	31, M	None	Brain abscess	N/A	Craniotomy/excision + CTX (7) + AMPC (28)	No relapse	[10]
11	37, M	CKH, PD	Peritonitis	PD catheter	CAZ + VCM (14)	Device removal	[11]
12	12, M	None	Endophthalmitis	N/A	CAZ^†^ + VCM^†^ + CEZ (5)	No relapse	[12]
13	33, F	SLE, PD	Peritonitis	PD catheter	CAZ + CEZ (14)	VCM (28) + device removal	[13]
14	6, M	ALL, FN	CRBSI	Hickman catheter	TAZ/PIPC + VCM (N/A)	No relapse	[14]
15	48, F	Breast cancer	CRBSI	Port-a-cath	CPFX (20)	TEIC (7) + device removal, LZD (7)	[15]
16	8, F	AML, FN	CRBSI	PICC	MEPM (19) + VCM (19) + device removal	No relapse	Our case

∗Child. †Intravitreal injection. ‡Duration of the treatment. AIDS, acquired immunodeficiency syndrome; ALL, acute lymphoblastic leukemia; AML, acute myeloid leukemia; AMPC, amoxicillin; CAZ, ceftazidime; CEZ, cefazolin; CEX, cephalexin; CKH, congenital kidney hypoplasia; CPFX, ciprofloxacin; CRBSI, catheter-related bloodstream infection; CTX, cefotaxime; CVA/AMPC, clavulanate/amoxicillin; CVC, central venous catheter; F, female; FN, febrile neutropenia; HD, hemodialysis; LZD, linezolid; M, male; MEPM, meropenem; MFLX, moxifloxacin; N/A, not available; NHL, non-Hodgkin's lymphoma; PD, peritoneal dialysis; PH, pulmonary hypertension; PIPC, piperacillin; Ref, reference; SLE, systemic lupus erythematosus; TAZ/PIPC, tazobactam/piperacillin; TEIC, teicoplanin; TOB, tobramycin; y, year; VCM, vancomycin.

## Data Availability

No data were used to support this study.
